# Animal Models for COVID-19: More to the Picture Than ACE2, Rodents, Ferrets, and Non-human Primates. A Case for Porcine Respiratory Coronavirus and the Obese Ossabaw Pig

**DOI:** 10.3389/fmicb.2020.573756

**Published:** 2020-09-25

**Authors:** Peter M. H. Heegaard, Michael Sturek, Mouhamad Alloosh, Graham J. Belsham

**Affiliations:** ^1^Department of Health Technology, Technical University of Denmark (DTU), Lyngby, Denmark; ^2^Department of Anatomy, Cell Biology, and Physiology, Indiana University School of Medicine, Indianapolis, IN, United States; ^3^Department of Veterinary and Animal Sciences, University of Copenhagen, Copenhagen, Denmark

**Keywords:** COVID-19, animal model, metabolic syndrome, Ossabaw pig, SARS-CoV-2

## Abstract

The ongoing COVID-19 pandemic caused by infection with SARS-CoV-2 has created an urgent need for animal models to enable study of basic infection and disease mechanisms and for development of vaccines, therapeutics, and diagnostics. Most research on animal models for COVID-19 has been directed toward rodents, transgenic rodents, and non-human primates. The primary focus has been on the angiotensin-converting enzyme 2 (ACE2), which is a host cell receptor for SARS-CoV-2. Among investigated species, irrespective of ACE2 spike protein binding, only mild (or no) disease has occurred following infection with SARS-CoV-2, suggesting that ACE2 may be necessary for infection but is not sufficient to determine the outcome of infection. The common trait of all species investigated as COVID models is their healthy status prior to virus challenge. In contrast, the vast majority of severe COVID-19 cases occur in people with chronic comorbidities such as diabetes, obesity, and/or cardiovascular disease. Healthy pigs express ACE2 protein that binds the viral spike protein but they are not susceptible to infection with SARS-CoV-2. However, certain pig breeds, such as the Ossabaw pig, can reproducibly be made obese and show most aspects of the metabolic syndrome, thus resembling the more than 80% of the critically ill COVID-19 patients admitted to hospitals. We urge considering infection with porcine respiratory coronavirus of metabolic syndrome pigs, such as the obese Ossabaw pig, as a highly relevant animal model of severe COVID-19.

## Introduction

In view of the ongoing SARS-CoV-2/COVID-19 pandemic (close to 20 million cases and more than 700,000 deaths worldwide at the time of writing; Johns Hopkins Corona Resource Center, 2020), there is an urgent need to increase the understanding of basic disease mechanisms, expand treatment possibilities, and identify biomarkers that correlate with the onset, severity, and duration of the disease. This will make it possible to reduce the disease complications for the individual patient and for societies worldwide despite the absence of general immunity toward COVID-19, be it created by efficient and comprehensive vaccination with yet to be developed vaccines or by herd immunity caused by the pandemic itself.

Animal models that faithfully reproduce human COVID-19 (i.e., incorporating the most important disease mechanisms, clinical signs, and response to treatment) are of utmost importance in order to achieve this. As commented by others (Callaway, 2020; Cleary et al., 2020; Cohen, 2020) animal species ranging from mice to non-human primates are actively being investigated in the quest for reproducible and faithful models of COVID-19.

## The ACE2 Receptor: Species Specific Differences in SARS-CoV-2 Spike Protein Binding and the Relevance for Animal Models

A specific focus has been on the angiotensin converting enzyme type 2 (ACE2) that is known to be a receptor for SARS-CoV (the original 2002–2003 coronavirus), and necessary for SARS-CoV to infect its host (Li et al., 2003). The affinity between the SARS-CoV spike protein and ACE2 has been found to be a major determinant for the susceptibility of the host to SARS-CoV infection (Wan et al., 2020). This suggested ACE2 as a possible host receptor also for SARS-CoV-2. ACE2 was indeed identified as a primary factor in determining cellular uptake of SARS-CoV-2 into engineered HeLa cells expressing ACE2 from different species (Zhou et al., 2020). The ACE2 from human, bat, pig, and civet but not from mouse, supported cellular uptake of SARS-CoV-2 (Zhou et al., 2020). Species-specific differences in the ability of ACE2 to bind the SARS-CoV-2 spike protein and thus in supporting virus entry was also predicted *in silico* from the ACE2-binding site of SARS-CoV spike protein (Luan et al., 2020; Wan et al., 2020). In most – but not all – cases, a predicted or experimentally proven high affinity between SARS-CoV-2 and the ACE2 protein correlates with susceptibility of the species in question to SARS-CoV-2 infection, although this does not always translate into disease (Table 1). As noted above, the predicted inability of mouse and rat ACE2 to bind SARS-CoV-2 (Wan et al., 2020) was demonstrated experimentally (Zhou et al., 2020) and, in accord with this, mice have been shown not to support SARS-CoV-2 replication and disease development (Bao et al., 2020). Using engineered (transgenic) mice harboring the human ACE2 receptor, virus propagation and development of mild disease has been demonstrated (Bao et al., 2020; Table 1). In addition to transgenic expression, human ACE2-expression in mice can be achieved by CRISPR mediated humanization of the mouse receptor or by viral vector-mediated expression of the human protein (using an engineered adenovirus; Cohen, 2020). Other SARS-CoV-2 infection models are based on existing SARS-CoV models such as the Syrian hamster in which SARS-CoV-2 has been reported to propagate and cause respiratory disease (Chan et al., 2020) although only “mild SARS-CoV-2 infection” was obtained (Sia et al., 2020). Ferrets are well-known models for influenza and also get infected with SARS-CoV-2; they express a spike protein-binding ACE2 protein, however only moderate disease is obtained (Kim et al., 2020). Also, farmed mink have been observed to be easily infected with SARS-CoV-2 but again infections are generally mild (Oreshkova et al., 2020). Non-human primates are an obvious choice based on closeness to humans genetically and a study reporting asymptomatic lung pathology after SARS-CoV-2 infection in cynomolgus macaques has appeared (Rockx et al., 2020). Similarly, mild symptoms in rhesus macaques with weight loss and signs of pneumonia but no fever were observed following SARS-CoV-2 inoculation (Munster et al., 2020; Shan et al., 2020). Such results again stress that a SARS-CoV-2 spike protein binding ACE2 receptor is necessary for virus entry into cells but may not be sufficient for obtaining more than mild COVID-19.

**Table 1:** Susceptibility to SARS-CoV-2 infection, disease signs, and ability of ACE2 to bind SARS-CoV-2 spike protein as predicted (based on protein sequence) or shown experimentally for various species.

Species	Susceptible to SARS-CoV-2 infection	Disease signs	ACE2 ‐ SARS-CoV-2 spike protein binding demonstrated experimentally (exp) or predicted (pred)
Pig, *Sus scrofa*	No (2,9)	No (2,9)	Yes (exp) (1)
Cat, *Felis catus*	Yes (2)	Yes, especially young cats (2)	Yes (pred) (3)
Dog, *Canis lupus familiaris*	Yes/minor (2)	No (2)	Yes (pred) (3)
Mouse, *Mus musculus*	No (5)	No (5)	No (exp) (1)
*Mus musculus* human ACE2 transgene	Yes (5)	Yes, mild (5)	Yes (exp) (5)
Ferret, *Mustela putorius furo*	Yes/upper resp. tract (2,9)	Yes, mild (2)	Yes (pred) (7)
Cynomolgus macaque, *Macaca fascicularis*	Yes (6)	Mild (6)	Yes (pred) (7)
Rhesus macaque, *Macaca mulatta*	Yes (8)	Mild (8)	Yes (pred) (7)
Masked palm civet, *Paguma larvata*	nd	nd	Yes (exp) (1) unfavorable (pred) (7)
Chinese horseshoe bat, *Rhinolophus sinicus*	nd	nd	Yes (exp) (1)
Greater horseshoe bat, *Rhinolophus ferrumequinum*	nd	nd	No (pred) (3)
Egyptian fruit bat, *Rousettus aegyptiacus*	Yes(9)	nd	nd
Chicken and ducks	No (2)	No (2)	nd
Pangolin, *Manis pentadactyla*	nd	nd	Yes (pred) (3)
Golden Syrian Hamster, *Mesocricetus auratus*	Yes (4)	Yes (4)	Yes (pred) (3)
Guinea pig, *Cavia porcellus*	nd	nd	No (pred) (3)

(1) Zhou et al. (2020); (2) Shi et al. (2020); (3) Luan et al. (2020); (4) Chan et al. (2020); (5) Bao et al. (2020); (6) Rockx et al. (2020); (7) Wan et al. (2020); (8) Munster et al. (2020); (9) Schlottau et al. (2020); nd: no data. The table reports on predicted spike protein ACE2 binding only if the interaction has not been shown experimentally.

The pig is an interesting case, as its ACE2 protein was both predicted (Wan et al., 2020) and demonstrated experimentally (Zhou et al., 2020) to bind SARS-CoV-2 spike protein; however SARS-CoV-2 neither replicated nor caused disease in pigs after experimental infection (Schlottau et al., 2020; Shi et al., 2020).

## Animal Models of Severe COVID-19

Thus, currently, no animal model of severe COVID-19 that reproduces the salient clinical and pathological features of the disease has been described. Mild symptoms are achievable in permissive species, but no model reproducing severe COVID-19, characterized by both upper and lower respiratory tract infection, pro-inflammatory cytokine activation, and prolonged disease is available, although such models obviously are of particular interest for investigation of intervention modalities and for biomarker research.

No clear correlation between ACE2 polymorphisms and COVID-19 susceptibility can be established (Devaux et al., 2020) and generally, the receptor recognition pattern of coronaviruses is complex and involves one or two domains of the spike protein, and both protein and carbohydrate host receptors (Li, 2015). Receptors include dipeptidyl peptidase 4 (receptor for MERS-CoV) and aminopeptidase N (APN), a receptor for the porcine respiratory coronavirus (PRCV) spike protein. In addition, host proteases are necessary for activating entry through concerted cleavage of the spike protein. Specifically, the spike protein of SARS-CoV-2 contains a cleavage site for the host protease furin (Coutard et al., 2020), which is essential for viral infectivity (Hoffmann et al., 2020). Cleavage by furin has to be followed by cleavage by transmembrane protease serine 2 (TMPRSS2) in order to fully activate the SARS-CoV-2 spike protein, which is similar to the mechanisms used by MERS-CoV (Hoffmann et al., 2020).

Observations in SARS-CoV-2-infected human subjects show that the disease spectrum of this infection is exceptionally wide, ranging from those with mild symptoms in the majority of cases (>80%) to severe, prolonged disease with high mortality and acute need for intensive care to alleviate symptoms and support survival (typically less than 5% of cases). A high proportion of the most severe cases occurs in patients with underlying chronic disease conditions (Deng et al., 2020; Zhou et al., 2020); depending on the region, 60–80% of COVID-19 patients requiring intensive care have at least one underlying co-morbidity [CDC COVID-19 Response Team (A), 2020; Tian et al., 2020]. In 5,700 COVID-19 patients admitted to New York hospitals, 88% had two or more comorbidities, the three most prevalent being hypertension (57%), obesity (42%), and diabetes (34%; Richardson et al., 2020). Also, case fatality rates are elevated in patients with pre-existing comorbidities such as cardiovascular disease, diabetes, chronic respiratory disease, hypertension, and cancer (5–10% compared to overall 2.3% among approx. 40,000 Chinese cases; Wu and McGoogan, 2020). Indeed, patients with type 2 diabetes and metabolic syndrome have been estimated to have up to 10 times greater risk of death when suffering from COVID-19 (Bornstein et al., 2020). Also, age, which is frequently associated with a metabolic syndrome-like state is an important risk factor for severe COVID-19. In the United States, as registered by mid-March 2020, of 4,226 COVID-19 cases 31% were in the above 65 years age group and this age group also accounted for 53% of intensive care unit admissions, and 80% of deaths associated with COVID-19 [CDC COVID-19 Response Team (B), 2020].

Cytokine storm in the lungs and inflammation are suggested as essential for the escalating and prolonged lung disease observed in severely affected COVID-19 patients, as is also the case for other severe human coronavirus infections like SARS and MERS (Mehta et al., 2020). Intriguingly, immunosuppressive intervention, as often applied to treat pro-inflammatory cytokine over-activation may, in fact, increase COVID-19 severity, depending on the timing and specificity of the treatment (Ritchie and Singanayagam, 2020). This corresponds to observations on infections with animal coronaviruses such as PRCV (Jung et al., 2007).

Since the majority of severe COVID-19 cases are characterized by having underlying chronic conditions, animal models incorporating such pre-existing conditions are needed to reproduce severe COVID-19. While other aspects of the infection may be studied in any susceptible animal model, including genetically modified animal models, the study of onset, development and resolution of severe COVID-19 in the background of existing disease may only be performed in an animal model that can reliably reproduce such disease states.

## The PRCV-Infected Obese Pig as an Animal Model of Severe COVID-19

There have been at least two, independent, but unsuccessful attempts to infect pigs with SARS-CoV-2 (Schlottau et al., 2020; Shi et al., 2020). However, a range of other coronaviruses do infect pigs (Saif, 2004; Saif and Jung, 2020; Yang et al., 2020), including porcine epidemic diarrhea virus (PEDV) and porcine respiratory coronavirus (PRCV). As mentioned above, PRCV utilizes another cellular receptor than SARS-CoV-2 for the spike protein mediated cell entry, namely APN (Delmas et al., 1993). However, as the pulmonary pathology of PRCV infection in pigs resembles SARS in humans PRCV infection of pigs was previously suggested as a model to examine SARS (Jung et al., 2007). Immunosuppression by corticosteroids (Jung et al., 2007; Zhang et al., 2008) or by co-infection with the immunosuppressive porcine respiratory and reproductive syndrome virus (PRRSV; Renukaradhya et al., 2010) were both demonstrated to exacerbate disease after PRCV infection into a SARS-like condition, with lower respiratory tract infection and pro-inflammatory cytokine activation. The pathogenesis due to PRCV infection also shows many of the same features as seen with SARS-CoV-2 infection (Saif and Jung, 2020), including a similar tissue tropism (upper and lower respiratory tract) and lung cellular tropism (type 1 and type 2 pneumocytes), and overlapping, although milder, clinical signs and lesions (fever, atypical pneumonia and interstitial pneumonia). Aspects lacking in non-complicated PRCV disease in comparison to COVID-19 include acute respiratory distress syndrome (ARDS), multiple organ failure and diffuse alveolar damage, as well as blood inflammatory responses and pulmonary fibrosis (Saif and Jung, 2020). Thus, respiratory disease due to non-complicated PRCV infection in pigs is a mild, self-limiting disease, similar to COVID-19 in most SARS-CoV-2 infected individuals (see above).

In line with this, we suggest that severe COVID-19 may be faithfully reproduced in PRCV-infected pigs co-affected by an underlying, chronic condition such as obesity associated metabolic syndrome. In addition to being an important comorbidity in the exacerbation of COVID-19 (see above), this syndrome has the dual characteristics of a pro-inflammatory state and immunosuppression (Andersen et al., 2016); both of these states are speculated to promote severe COVID-19 (Ritchie and Singanayagam, 2020).

We would like to emphasize that in addition to closely mirroring human physiology, anatomy, immunology and food preferences, a number of pig breeds have been extensively documented to be an excellent model for human diet-induced obesity including the spectrum of inflammation-related co-morbidities associated with this condition (metabolic syndrome; Sturek et al., 2020). This is especially true for the Ossabaw pig that has been described as having the greatest capacity of any known, terrestrial mammal to produce and store fat (Brisbin and Mayer, 2001). We, and others, have documented that high energy and high fat diets result in the rapid occurrence of obesity in Ossabaw pigs (Sturek et al., 2020). This reproducibly leads to a metabolic syndrome state with clinical signs such as hypertension, high fasting blood glucose, and dyslipidemia, with more advanced end-points such as pre-diabetes, non-alcoholic steatohepatitis (NASH), and cardiovascular disease routinely achieved. These conditions are accompanied by signs of a systemic inflammatory state with increased circulatory biomarkers of inflammation (Rødgaard et al., 2013).

As described above, the pig is the natural host of a number of well-described coronavirus infections with various tropisms and virulence. None of these viruses are human pathogens. PRCV has been demonstrated, both by intranasal and intratracheal instillation, to lead to respiratory disease in pigs, although with low morbidity and mortality (Saif, 2004). As also described above, the outcome of the infection was worsened by immunosuppression with dexamethasone (Jung et al., 2007; Zhang et al., 2008). We hypothesize that disease severity will increase in obese Ossabaw pigs infected with PRCV compared to pigs of normal weight, and hence will constitute a useful model for severe COVID-19 in humans at risk due to metabolic syndrome associated comorbidities, including aged individuals. With the added benefit of being a well-described pig-specific virus (with no rigorous biosafety demands), we suggest that the obese pig affected by the metabolic syndrome will constitute a highly human-translatable animal model having the potential to significantly facilitate and accelerate SARS-CoV-2/COVID-19 research. Both basic disease mechanisms and the efficacy and safety of therapeutic drug candidates for treatment of COVID-19 patients from high risk groups can potentially be studied in this model as can biomarkers for severe COVID-19; such markers are of high importance to identify individuals facing severe disease progression to allow early treatment decisions.

## Discussion

In summary, there is a compelling need for animal models for COVID-19 that accurately reproduce severe COVID-19, as these human cases are currently non-treatable and have resulted in very severe societal measures having detrimental economic and political consequences worldwide. While a range of animal species are indeed permissive for SARS-CoV-2 infection, only mild or no disease is obtained upon infection. We suggest that a relevant and valid model of severe COVID-19 should preferentially incorporate the most common comorbidities of severe COVID-19, such as metabolic syndrome, type 2 diabetes, and hypertension, also associated with age. We propose that the persistent inflammation that is part of the metabolic syndrome increases the risk of a cytokine storm during coronavirus infection linking an aberrant metabolic state to severe COVID-19.

Non-human primates may be highly relevant as models for SARS-CoV-2 infection, however developing primate comorbidity models will be expensive and time-consuming. Pig models are highly relevant to human medicine because they closely mimic human anatomy, physiology, and immunology. Furthermore, they develop robust metabolic syndrome and cardiorespiratory disease upon diet-induced obesity. Intranasal inoculation of PRCV in pigs with robust metabolic syndrome such as obese Ossabaw miniature pigs, may increase the severity of disease, similar to patients with metabolic syndrome having severe COVID-19. This type of animal model could accelerate coronavirus research on basic disease mechanisms, biomarkers, and therapeutics for COVID-19 patients in high-risk groups.

## Author Contributions

The review was written by all authors with PH as lead. All authors contributed to the article and approved the submitted version.

### Conflict of Interest

MS and MA are cofounders of CorVus Biomedical, LLC.

The remaining authors declare that the research was conducted in the absence of any commercial or financial relationships that could be construed as a potential conflict of interest.
